# Rubinstein-Taybi syndrome with scoliosis

**DOI:** 10.1186/1748-7161-6-21

**Published:** 2011-09-30

**Authors:** Yasunori Tatara, Noriaki Kawakami, Taichi Tsuji, Kazuyoshi Miyasaka, Tetsuya Ohara, Ayato Nohara

**Affiliations:** 1 Gunma Spine Center, Harunaso Hospital, Takasaki, Gunma, Japan; 2 Meijo Hospital, Spine Center, Nagoya, Aichi, Japan

**Keywords:** Rubinstein-Taybi syndrome, scoliosis, surgery

## Abstract

**Study Design:**

Case report.

**Objective:**

The authors present the case of a 14-year-old boy with Rubinstein-Taybi syndrome (RSTS) presenting scoliosis.

**Summary of Background Data:**

There have been no reports on surgery for RSTS presenting scoliosis.

**Methods:**

The patient was referred to our hospital for evaluation of a progressive spinal curvature. A standing anteroposterior spine radiograph at presentation to our hospital revealed an 84-degree right thoracic curve from T6 to T12, along with a 63-degree left lumbar compensatory curve from T12 to L4. We planned a two-staged surgery and decided to fuse from T4 to L4. The first operation was front-back surgery because of the rigidity of the right thoracic curve. The second operation of lumbar anterior discectomy and fusion was arranged 9 months after the first surgery to prevent the crankshaft phenomenon due to his natural course of adolescent growth. To avoid respiratory complications, the patient was put on a respirator in the ICU for several days after both surgeries.

**Results:**

Full-length spine radiographs after the first surgery revealed no instrumentation failure and showed that the right thoracic curve was corrected to 31 degrees and the left lumbar curve was corrected to 34 degrees. No postoperative complications occurred after both surgeries.

**Conclusions:**

We succeeded in treating the patient without complications. Full-length spine standing radiographs at one year after the second operation demonstrated a stable bony arthrodesis with no loss of initial correction.

## Background

Rubinstein-Taybi syndrome (RSTS) is characterized by mental retardation and physical abnormalities such as broad thumbs and halluces, short stature, and a peculiar facial expression - 'comical face' which is characterized by a beaked nose, down-slanting palpebral fissures and hypoplastic maxilla [1-5]. There have been several reports [6-15] on surgical intervention to treat congenital dislocation of the patella or thumb deformity in patients with RSTS, but we have yet to see case reports on surgical procedures to correct scoliosis. In this report, we present an operated case of an RSTS patient with scoliosis whose spine was successfully realigned and balanced.

## Case presentation

### History and Examination

**Figure 1:**
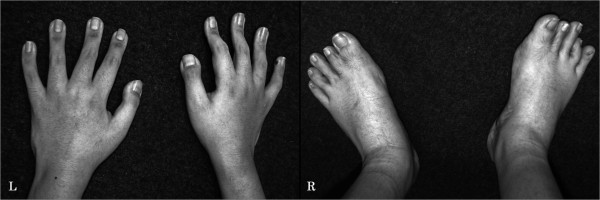
**Photographs of the patient showing his broad thumbs (left) and halluces (right)**.

**Figure 2:**
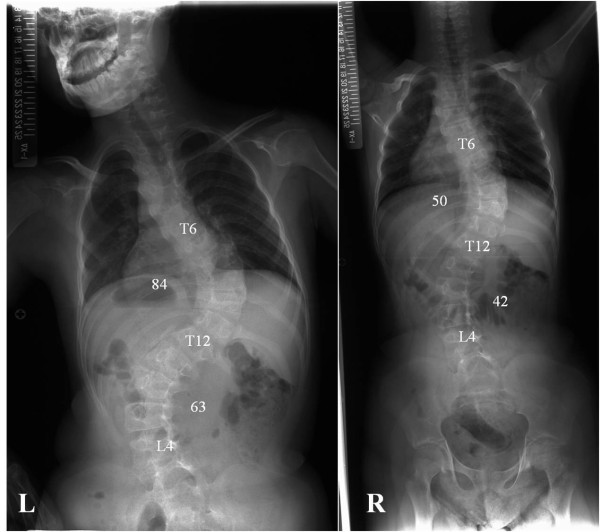
**Preoperative full-length spine radiographs**. Left: standing, Right: traction.

A 14-year-old boy was referred to our hospital for evaluation of a progressive spinal curvature. He had already been diagnosed with RSTS and noted to have spinal deformity at age one in a regional hospital. Since then he had undergone brace treatment, which failed to halt progression of the scoliosis. There was no mention of either RSTS or scoliosis in his family history. He manifested a peculiar facial appearance, broad thumbs, broad halluces, short stature, and mental retardation (Figure 1 L and R). He was unable to walk without aid, but did not manifest any neurological symptoms such as hyperreflexia or abnormal abdominal reflexes. His general condition was thoroughly examined for any other anomalies before surgery, and we found no airway problems or congenital heart disease. Physical examination revealed a marked right thoracic rib prominence, right shoulder elevation, asymmetric scapulae, and pelvic obliquity. A standing anteroposterior spine radiograph at presentation to our hospital revealed an 84-degree right thoracic curve from T6 to T12, along with a 63-degree left lumbar compensatory curve from T12 to L4. Additional full-length spinal side bending and traction radiographs were obtained to evaluate curve flexibility. On the traction film, the right thoracic curve and the left lumbar curve were corrected to 50 and 42 degrees, respectively (Figure 2 L and R). At the time of surgery, he was 135 centimeters tall and weighed 32 kilograms, and his secondary sex characteristic had not emerged yet. The preoperative films revealed that the triradiate cartilage was open and the Risser grade was 4.

### Operation

We planned a two-staged surgery. The first operation was front-back surgery because of the rigidity of the right thoracic curve. The second operation, a lumbar anterior discectomy and fusion, was arranged 9 months after the first surgery in order to prevent a crankshaft phenomenon induced by his natural growth.

### First operation

The patient was laid in the left lateral decubitus position. We partially resected the right 8th rib and entered the pleural cavity. Anterior discectomy from T8/9 to T10/11 was performed, and we used the resected rib as a bone graft in that region. A chest tube was inserted into the pleural cavity. Immediately after the anterior surgery, halo-femoral traction was applied to facilitate the posterior surgery, and then the patient was turned to the prone position. Smith-Petersen osteotomy was performed from T8/9 to T10/11, followed by a posterior corrective fusion and instrumentation with a pedicle screw system from T4 to L4. We used the combination of an allograft and local bone graft in the instrumented region. Preoperative evaluation revealed that the lumbar curve could be manually corrected to only 42 degrees, and the inclination of L4 in the coronal plane was residual, so we extended the fusion to L4. Transcranial electrical stimulation motor-evoked potential monitoring showed no significant intraoperative change in potentials. The operation time was 421 minutes and the estimated blood loss was 2240 ml. No additional transfusion was needed because of the autologous blood banked before surgery and because an intraoperative cell saver was used. Full-length spine standing radiographs at 2 weeks after surgery revealed no instrumentation failure and showed that the right thoracic curve from T6 to T12 was corrected to 31 degrees and the left lumbar curve from T12 to L4 was corrected to 34 degrees.

### Second operation

The patient was laid in the right lateral decubitus position. We used the anterior retroperitoneal approach by resecting the left 11th rib, and then performed a discectomy from L1/2 to L3/4 was performed. A combination of the risected rib and hydroxyapatite spacers was used for bone grafting in that region. The operation time was 146 minutes and the estimated blood loss was 500 ml.

### Postoperative course

**Figure 3:**
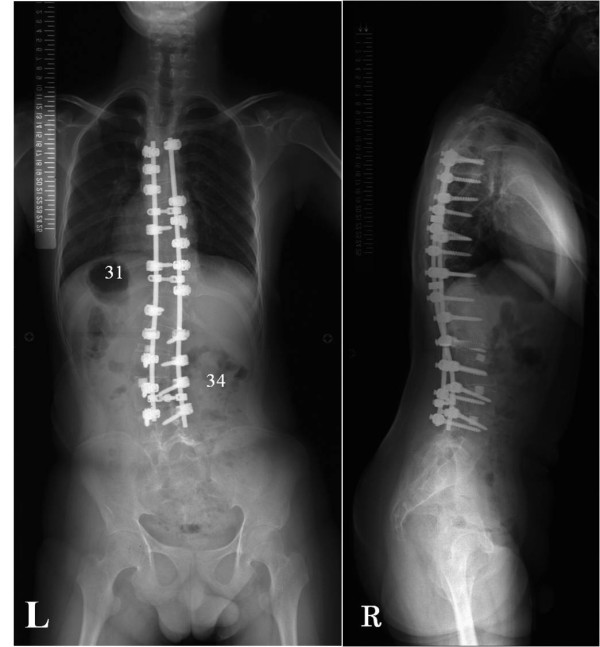
**Full-length spine standing radiographs at one year after the second operation**. The initial correction is maintained. The Cobb angle from T6 to T12 is 31 degrees and the angle from T12 to L4 is 34 degrees. Left: coronal, Right: sagittal.

He entered the ICU immediately after both the first and second surgeries and was maintained on a respirator for several days in order to prevent respiratory complications. The patient wore a thoracolumbar spinal orthosis for 6 months after the first surgery. Full-length spine standing radiographs at one year after the second operation demonstrated a stable bony arthrodesis with no loss of initial correction (Figure 3 L and R). His parents consented to submitting data from the case for publication.

## Discussion

RSTS is a rare human genetic disorder [16] and is associated with well-defined multiple congenital anomalies. It generally occurs sporadically, and equally affects males and females. The birth prevalence is 1 in 100, 000 to 125, 000 [17]. The diagnosis is essentially based on clinical manifestations; however, evaluation for a microdeletion at chromosome 16p13.3 and mutations in CBP (cAMP-responsive element binding protein binding protein) and p300 is helpful in diagnosing when an abnormality is found [18].

Wiley [19] made a full report on various medical problems in RSTS. Among these prob-lems, airway anomalies [20] such as anterior larynx [21] and easy collapsibility of the laryngeal wall, congenital heart disease (24-38%), gastroesophageal reflux, and renal anomalies (52%) may possibly become major perioperative problems. Surgeons should also be aware of the high occurrence of skin trouble such as keloids and hypertrophic scars in RSTS. Therefore, we have to carefully examine the general condition of RSTS patients before surgery.

There have been several reports of surgery on RSTS patients but no reports of surgery on scoliosis have been published. Limited to orthopedic or spine surgery, there were 5 reports of knee surgery for patella dislocation [8-10,13,14], 3 reports of hand surgery for thumb deformity [7,11,15], one report of foot surgery for postaxial polydactyly [4], one report of cervical spine surgery for myelopathy [6], and one report of lumbar spine surgery for tethered spinal cord [12]. None of these reports mentioned any perioperative complications in these reports in spite of various medical problems in RSTS.

We succeeded in treating the patient without complications even though two major surge-ries were performed. The first surgery was front-back surgery with thoracotomy, and the second surgery was performed using retroperitoneal approach with a rib resection. We had to pay the closest attention to the patient whose mental retardation prevented him from adequately complaining of any symptoms. Therefore, in order to prevent any respiratory complications, we judged that the patient should be maintained on a respirator in the ICU after both surgeries. Consequently, no postoperative problems oc-curred.

The crankshaft phenomenon is a complication known to occur after spinal fusion in skeletally immature patients with scoliosis. When asymmetric anterior growth continues after solid posterior fusion, it causes the crankshaft phenomenon. Risser sign, Tanner stage, triradiate cartilage, peak height velocity, and chronologic age have all been used as elements to predict the occurrence of a crankshaft in idiopathic scoliosis [22-27].

The preoperative films revealed that the triradiate cartilage was open and the Risser grade was 4. His secondary sex characteristic had not emerged yet at the time of surgery even though he was 14 years old. The patient's growth spurt had not reached its peak height velocity, but still he had a large secondary lumbar curve. We had discussed extensively with the parents about the importance and necessity of a second operation. The parents feared that even if the crankshaft phenomenon caused correction loss of the lumbar curve, the patient's mental retardation might possibly prevent a manifestation of clear symptoms. Therefore, in order to minimize the possibility of a crankshaft, we performed a secondary procedure of anterior lumbar discectomy and fusion. As a result, x-ray films at one year after second surgery demonstrated a stable bony arthrodesis with no loss of initial correction.

Rubinstein [28] reported that children with RSTS could have congenital or acquired scoliosis, kyphosis, or lordosis. The patient in this case report was diagnosed with scoliosis at the young age of one and was still unable to walk without aid. Therefore, we believe that scoliosis in RSTS patients may be caused by associated neuromuscular abnormalities, and not simply by accidental reasons.

## Conclusions

We succeeded in treating the patient without complications. Full-length spine standing radiographs at one year after the second operation demonstrated a stable bony arthrodesis with no loss of initial correction. Spinal disorders in RSTS patients must be carefully assessed and treated if necessary.

## Consent

Written informed consent was obtained from the parents of the patient for publication of this case report and any accompanying images. A copy of the written consent is available for review from the Editor-in-Chief of this journal.

## Competing interests

The authors declare that they have no competing interests.

## Authors' contributions

YT conceived the study and drafted the manuscript; NK performed surgeries, and performed critical revision of the manuscript and gave final approval of the version to be published; TT, KM, and AN performed surgeries, and collected and reviewed clinical and radiographic charts. All authors read and approved the final manuscript.
